# “Sharing is my only option”: an ethnographic analysis of the underlying contexts of needle and syringe sharing among people who inject drugs using the socio-ecological framework

**DOI:** 10.3389/fpubh.2025.1527307

**Published:** 2025-10-22

**Authors:** Mohammad Niaz Morshed Khan, Samira Dishti Irfan, Md. Alamgir Hossain, Michiko Moriyama, Sharful Islam Khan

**Affiliations:** ^1^Program for HIV and AIDS, Health Systems and Population Studies Division, International Centre for Diarrhoeal Diseases Research, Dhaka, Bangladesh; ^2^Graduate School of Biomedical and Health Sciences, Hiroshima University, Hiroshima, Japan; ^3^Maternal and Child Health Division, International Centre for Diarrhoeal Diseases Research, Dhaka, Bangladesh

**Keywords:** PWID, ethnography, needle and syringe sharing, HIV, Bangladesh

## Abstract

**Background:**

People who inject drugs (PWID) in Bangladesh are part of a long-standing needle syringe program, which faced challenges in containing the spread of HIV, thus warranting exploration of the drivers of needle and syringe sharing. This article aimed to explore the underlying reasons for needle and syringe sharing among PWID through ethnographic lenses in Dhaka.

**Methods:**

We adopted peer-driven Participatory Ethnographic and Evaluation Research, entailing 6,000 h of observations at service delivery points and drug-injecting spots, 66 in-depth interviews and seven focus groups with PWID and 29 key-informant interviews with service providers, program experts and policy stakeholders. Data were thematically analyzed as per the socioecological model.

**Results:**

The findings presented multilayered contexts driving needle and syringe sharing. At the intrapersonal level, PWID possessed myths and misconceptions regarding needle and syringe sharing, which hindered their risk perception of needle and syringe sharing. As many PWID, especially street-based PWID, felt despondence and distress toward their life, they perceived safe injecting as futile as they were already in fatal conditions. Some PWID partook in concurrent substance use, clouding their judgment, increasing their aggression, and perpetuating risky injection. Moreover, findings showed that withdrawal took precedence in needle and syringe sharing behaviors. At the interpersonal level, PWID communities protected fraternal relationships through sharing, inherited community-bred misinformation about safe injecting practices, and were influenced by gendered and community hierarchies and power dynamics within the PWID sub-culture, which all fueled needle and syringe sharing. Shadowing sessions, observations and interviews revealed challenges at the organizational level (i.e., the PWID intervention) such as inconvenient outreach schedules in relation to PWID's drug-injecting time windows; challenges in the needle and syringe distribution approach; and a predominantly peer-focused outreach approach where peers exhibited work performance and compliance issues, and challenges in capacity building and upholding motivation among OWs. At the structural level, changes in infrastructure and associated inconveniences, criminalization of drug use and harassment of PWID, increased drug prices and financial constraints, and changes in fund allocation policy of the donor engendered risky injection.

**Conclusion:**

The findings presented multifaceted reasons for needle and syringe sharing, thus warranting multilayered interventions. It is not possible to contain needle and syringe sharing and alleviate HIV epidemics through addressing proximate causes or structural interventions alone, thus efforts need to be invested in broadening the horizons for harm reduction interventions.

## 1 Introduction

Drug use remains a global health threat as one of three persons aged between 15 and 64 has used a drug within the past year as of 2023 (1). However, drug reports indicate that opioids contribute to the greatest harm among all drugs. A total of 60 million people engaged in non-medical opioid use. Estimates showed that 14.0 million people injected drugs (2). The East and Southeast Asian regions were shown to have the highest proportion of people who inject drugs (PWID) (2). For PWID, global health challenges have been attributed to the sharing of needles and syringes (3) including HIV and Hepatitis C. HIV, which stands for Human Immunodeficiency Virus, is a retrovirus that infects cells of the immune system and destroys their key functions (4). Whereas, Hepatitis C virus is an inflammatory liver disease which could lead to both acute and chronic hepatitis, potentially causing life-threatening illnesses such as cancer (5). Though HIV and HCV differ in structure and behavior, both viruses share a similar transmission pathway: direct bloodstream exposure via contaminated needles. Blood usually comes in contact with a mucous membrane or damaged tissue or is directly injected into the bloodstream, for the virus to take effect (6). However, the hepatitis C virus is about 10 times more concentrated in blood than HIV, meaning that the hepatitis C virus can be transmitted more easily through blood (7). On the other hand, the per-injection risk of transmission of HIV through a contaminated needle was estimated to be between 0.7% and 0.8% for PWID (8). HIV typically progresses throughout three stages: acute HIV infection (where the person exhibits flu-like symptoms), followed by chronic HIV infection (where the patient remains asymptomatic but the virus continues to reproduce throughout the body), then AIDS, which is the most severe stage of HIV infection which permanently damages the immune system and yields high viral load (6). If not treated with anti-retroviral therapy (ART), HIV could progress to a death sentence (5). On the other hand, the ultimate consequences of Hepatitis C virus include chronic liver inflammation and scarring, failure of liver and elevated risk of liver cancer, posing as a “silent killer” (9, 10). If preventive measures are not taken for sharing needles and syringes among PWID, the incidence of HIV and HCV will further increase, which could contribute to increased prevalence of both conditions for any country context (11). The HIV acquisition risk is 14 times higher for PWID than those who do not inject drugs (12). Moreover, one in eight PWID are living with HIV (1.7 million people or 12% of people who use drugs) and every second person is living with Hepatitis C (3, 12).

Notably, despite the implementation of needle and syringe programs (NSP) in 93 countries (13), global literature depicted high rates of needle and syringe sharing. A recent systematic review indicated that 32.8% of the PWID engaged in needle and syringe sharing within the past 6 months, of which the South Asian region demonstrated one of the highest percentages (32.1%) (14). A recent analysis in Pakistan revealed an unweighted HIV prevalence of 21.0% among PWID, which was significantly associated with receptive syringe sharing (15). Similarly, a systematic review in India identified a pooled prevalence of 7.8%−57.1% needle and syringe sharing, which was depicted as the most significant determinant of HIV prevalence among PWID (16). Although harm reduction interventions are established in these countries, the HIV burden persists. Moreover, PWID's exposure to risky injecting behaviors [which is defined as sharing (borrowing/lending) unsterile needles and syringes; and sharing injecting parapharnelia (17, 18)] and ill-health is amplified by concurrent drug use, predominantly methamphetamine. For instance, the lifetime and current prevalence of methamphetamine use among PWID in a study based in China and Myanmar was 84.2% and 75.2%, respectively (19). This concurrent drug use pattern was also corroborated by qualitative evidence in Vietnam (20) and in a systematic review among 28 countries in Asia and North America (21).

The same scenario resonates in Bangladesh, which has an established harm reduction (NSP) intervention since 1998 (22). Several rounds of Integrated Biological and Behavioral Surveillance (IBBS) among key populations (KPs) indicated a concentrated HIV prevalence among PWID. For instance, the most recent IBBS demonstrated 4.1% and 33.2% prevalence of HIV and HCV respectively in 2020 (23). According to recent national case reporting findings, PWID constitute 13.1% (i.e., 167 cases out of 12,076) of the total detected cases of HIV identified in 2023, which has increased compared to 11.0% of the total HIV caseload in 2022 (24). PWID also comprise of one of the highest caseloads among all of the key population groups in Bangladesh (25). Approximately a third (33.2%) of the PWID were living with HCV (23). Moreover, the recent round of IBBS indicated that less than a half of the PWID in the country have always used sterile needles when injecting within the past month (23).

This epidemiological situation demonstrated the need for an in-depth analysis of the underlying contexts of needle and syringe among PWID. Specifically, it is crucial to excavate the gaps and challenges of the existing needle and syringe program, to understand why the existing program modality is unable to stop sharing. However, alongside exploring the programmatic limitations, it is equally important to understand overarching socio-structural contexts driving needle and syringe sharing which harm reduction interventions struggle to address. Thus, a multifaceted analysis of this nature would foster a nuanced, contextualized understanding of the precipitants of needle and syringe sharing which could not only contribute to the current knowledge base, but also set the precedent for a structural, holistic intervention that accounts for various facets of PWID's lives in Bangladesh and other settings.

Though there is a growing body of quantitative and qualitative evidence about the correlates of needle and syringe sharing, they are mostly limited to common determinants without grounding it within a conceptual proposition. However, placing the analysis within a conceptual framework would set clear guidelines for future interventions. A few studies were conducted in Bangladesh about risky injecting behaviors of PWID (26–28). Although, the only other qualitative study was conducted in a region without a concentrated HIV epidemic which holds a different social context (27). However, an analysis of the underlying contexts of risky injection is urgently needed in epidemic epicenters. Thus, this article aimed to explore the reasons for needle and syringe sharing of PWID and increased HIV prevalence among these populations in Dhaka city, Bangladesh using peer ethnography.

### 1.1 Theoretical framework: socio-ecological model

Locally and globally, harm reduction interventions focus more on individual risk reduction, although PWID experience various socio-structural impediments including poverty, discrimination and homelessness (29, 30). This phenomenon aligns with the socio-ecological model by Bronfenbrenner (49) which examines how environmental, socio-political, legal, cultural, organizational, community and intrapersonal circumstances influenced human behaviors. In the HIV literature, this framework has been acclaimed for its ability to guide the assessment of risk contexts underlining HIV epidemics. While understanding the proximal risks are necessary for mediating HIV transmission, risks also need to be explored at socio-structural layers (31). Therefore, this analysis will apply the socio-ecological model to understand the contexts of needle and syringe sharing on the intrapersonal, interpersonal, organizational, and structural levels.

## 2 Materials and methods

### 2.1 Study design and sites

To attain an in-depth, contextual understanding of the individual and socio-structural challenges associated with needle and syringe sharing, we adopted the ethnographic design. In particular, we adopted the Participatory Ethnographic and Evaluation Research (PEER) approach to gain an intimate understanding of the perceptions and lived experiences of needle and syringe sharing through immersing into their daily culture (32). This approach is acclaimed for stimulating extended engagement with the study community (33–35), which may have not been possible through quantitative or traditional qualitative research designs (36). Alongside non-peer researchers, we deployed PWID community members as peer researchers and engaged them throughout various parts of the study.

Study sites were selected based on available evidence about areas with higher concentrations of HIV-positive PWID was high. We purposively selected six Drop-in Centers (DICs) and 98 drug-injecting venues i.e., spots/sub-spots proximal to the DICs (37). DICs are harm reduction service delivery points that administer various services within both the premises and drug-injecting spots. In DICs, PWID can uptake services including treatment for infection-related injuries, sexually transmitted infections including HIV and general health ailments. At drug-injecting spots, peer outreach workers distribute needles and syringes, condoms and provide behavior change communication sessions.

### 2.2 Data collection

Our inclusion criteria for this study included PWID of any gender and other research participants (such as service providers, key-informants described below, etc.) aged 18–60 years old, who provided their verbal (for PWID) and written (for non-PWID) informed consents. To be defined as a PWID for this study, one needed to have a history of injecting drugs within the last 6 months. Service providers include those who are working for the harm reduction interventions during the study period. Exclusion criteria comprised of participants who were <18 years old due to lack of availability and older than 60 years due to inability to concentrate on any discussion. These inclusion and exclusion criteria were followed for all components of the ethnography including IDIs, FGDs, KIIs, informal discussions, walk-and-talk sessions, and shadowing, more details have been provided in the respective sections.

Our research team consisted of four peer researchers (i.e. current and ex-PWID) and five non-peer researchers who were medical anthropologists, public health professionals and sociologists who previously worked in qualitative research projects with PWID. All of the researchers contributed to the observations. The peer researchers were already networked with their communities, and thus able to facilitate access of non-peer researchers into their communities. The role of peer researchers were not only limited to data collection. Before being deployed on the field, they underwent intensive training of 10 days in diverse ethnographic data collection techniques, research ethics, primary data collection (including field note-taking) and analysis strategies, mock interviewing and field testing, etc. Since their capacities were enhanced through prolonged training, they were able to partake in data analysis (including coding) and interpretation of findings. Their perspectives and lived experiences were also considered during the framing of our findings. This research protocol attained the ethical approval of the ethical review committees of the Institutional Review Board of the organization (PR-18014). As per the ethical guidelines of the organization, understood and informed consents were obtained from the study participants.

Our data collection encompassed a blend of ethnographic data collection approaches, including participatory observations (including shadowing, and walk-and-talk tours), in-depth interviews (IDIs), focus group discussions (FGDs) and key-informant interviews (KIIs). For participatory observations, we applied an observation matrix checklist for initial observations and an evolving checklist responding to field-level changes. Our participatory observations aimed to explore how their social interactions and lived realities governed their injection behaviors. Smaller research teams of both peer and non-peer researchers conducted observations, usually in roster shifts throughout different locations, in and around drug-injecting spots from the first to the last injecting episode from the early morning (5am) to night (12am), constituting a total of 6,000 h. These observation sessions spanned over weekdays, weekends, public holidays, and religious festivals (i.e., Eid). This ethnography adopted diverse observational techniques such as “shadowing” sessions (38) and “walk and talk” tours (35, 39). To attain an emic understanding of the realities constructing their needle and syringe sharing behaviors, we shadowed 18 PWID and 32 outreach workers during their working hours. The shadowing participants were selected based on their willingness, availability and drug-using and sharing history (which we found out through previous informal conversations). In the shadowing sessions, the outreach workers, who were also often members of the PWID community, worked in their assigned catchment areas for the harm reduction interventions for PWID as a staff of an NGO service center. Most of them were male. They were shadowed their outreach duty hours (morning to evening sessions). The duration of the shadowing sessions for each PWID varied, depending on their availability, comfort, level of intoxication/withdrawal, ranging from 2 to 3 h to a couple of days. The shadowing sessions explored their drug collection, injecting and needle and syringe and paraphernalia sharing practices (both borrowing and lending), community dynamics, interactions with the OWs, etc.

Whereas, we opted for the “walk-and-talk” tour approach with the PWID who we perceived would be able to vocalize their lived experiences and perspectives about the contexts of needle and syringe sharing. We also chose participants with prolonged history of injecting drug use and their association with the harm reduction program. We conducted a total of 52 walk-and-talk tours with PWID. Walk-and-talk sessions were conducted at drug-using spots, during their optimal drug-injecting time windows, which could range any time from early morning to late evening. Each session lasted 1–3 h, depending on the participants' comfort and availability. The issues investigated included: circumstances that compel them toward needle and syringe sharing, drug use, collection, and sharing practices, harm reduction uptake practices, relationships and dynamics with the PWID community, and lived experiences of structural harassment.

We also performed 100 informal interviews as it was not possible to document individual narratives exclusively through observations (40). Informal interviews took place during the onset of IDIs. If we observed any particular behaviors warranting further exploration, we approached both PWID and outreach workers for informal interviews. We inquired about issues such as non-programmatic and programmatic contexts driving needle and syringe sharing practices, and drawbacks of the existing harm reduction program. Each interview lasted 15–30 min. During observations, peer and non-peer researchers took brief notes, which were immediately expanded, either on the same day, or the next day. Depending on their technological literacy and access, researchers either opted for typing or writing field notes by hand. These fields were ultimately compiled into an ethnographic record, consisting of approximately 15,000 pages. Alongside taking field notes, we also took photographs or videos at the observations after taking permission of the participants. These visuals were also appended to the ethnographic record. The ethnographic record was analyzed using the data analysis techniques described below.

We conducted 66 IDIs with male, female and transgender (*hijra*) PWID, aged 18 to 60 years old, who injected drugs at least once in the last 6 months. To capture cross-cutting issues across different socio-demographic groups, we opted for maximum variation sampling for the IDIs (41). In this specific approach, we have applied maximum diversity sampling in terms of age, occupation, HIV status, needle and syringe sharing behaviors, gender, marital status and living status (home-based vs. street-based). The interview guides of the IDIs collected information such as socio-demographic characteristics, drug collection and using practices, reasons for needle and syringe sharing, experiences and feedback about the harm reduction intervention, harassment/violence lived experiences, myths and misconceptions about sharing, perceived reasons for the HIV increase, etc. Each IDI lasted for 45–60 min. While IDIs were able to elicit individual perspectives, beliefs and emotions about needle and syringe sharing, FGDs were beneficial for extracting socio-structural perspectives about needle and syringe sharing. Seven FGDs were conducted on six to nine PWID or service providers per group, using homogeneous sampling (41). FGDs were conducted on a total of 4 groups of PWID (30 PWID) and 3 groups of service providers (21 service providers including DIC managers, outreach supervisors, and experienced outreach workers). Each FGD lasted ~60–90 min. FGD interview guides included community perspectives of needle and syringe sharing, perceived limitations of the harm reduction program, lived experiences of harassment and violence (for PWID only), and recommendations for improving the harm reduction program. We conducted interviews in vacant rooms of DICs, secluded park corners, etc. We conducted 29 KIIs with information-rich groups including harm reduction program experts, policymakers, psychiatrist, experienced DIC staff and leaders of community-based organizations. We believed that conducting KIIs on these information-intense groups would elicit perspectives from the larger structural/policy layers which may not have been covered otherwise. KII participants were selected through intensity sampling and snowball sampling techniques (41). Interview guides for KIIs included issues such as challenges and drawbacks of the harm reduction program, programmatic reasons for needle and syringe sharing, and the perceived reasons for the HIV increase, and recommendations to improve the existing harm reduction approach. Each interview lasted 45–60 min.

### 2.3 Data analysis

We commenced data analysis while data collection was ongoing, until we reached data saturation. When we noticed that themes were repeating, we were assured that we have captured the full range of practices, beliefs and contextual nuances surrounding needle and syringe sharing (42).

The interview and observation guidelines were consistently modified throughout data collection and analysis, depending on emerging field issues. Our researchers thematically categorized and sorted data as per the layers of the socio-ecological model, which are both conceptual and cognitive processes that do not warrant software (43). Rather, qualitative scholars have identified a few key limitations of software which include: the loss of nuanced contextualization and holistic understanding of the participants' narratives; their tendency to over-code rather than interpret; the risk for inconsistent coding; and reductionism in meaning of sensitive anecdotes (44, 45).

This study applied thematic analysis approaches as per Braun and Clarke's six steps of thematic analysis (46). After sorting, organizing, and coding the interview and observational data, we categorized the reasons for needle and syringe sharing into the intrapersonal, interpersonal, organizational, and structural layers, and eventually developed a codebook. We applied collaborative coding to pinpoint major themes and sub-themes and their contextual meanings. Inter-coder reliability was maintained by formulating a unanimous interpretation before collating a code and resolving inter-coder discordances (47). Although most coding decisions were unanimous, there were 11 disagreements, which were resolved by senior team members. Decision trails were documented throughout data analysis.

To ensure scientific rigor, specific methodologies were employed as per qualitative expert recommendations. The use of various data collection techniques, researchers, analytical tools and perspectives helped enhance data triangulation (41). Peer debriefing sessions, which engaged peer and non-peer researchers helped strengthen the emic understanding of needle and syringe sharing contexts. We also conducted member-checking sessions with the PWID by sharing preliminary findings and asking for feedback about our interpretative findings (48).

## 3 Results

**Figure 1:**
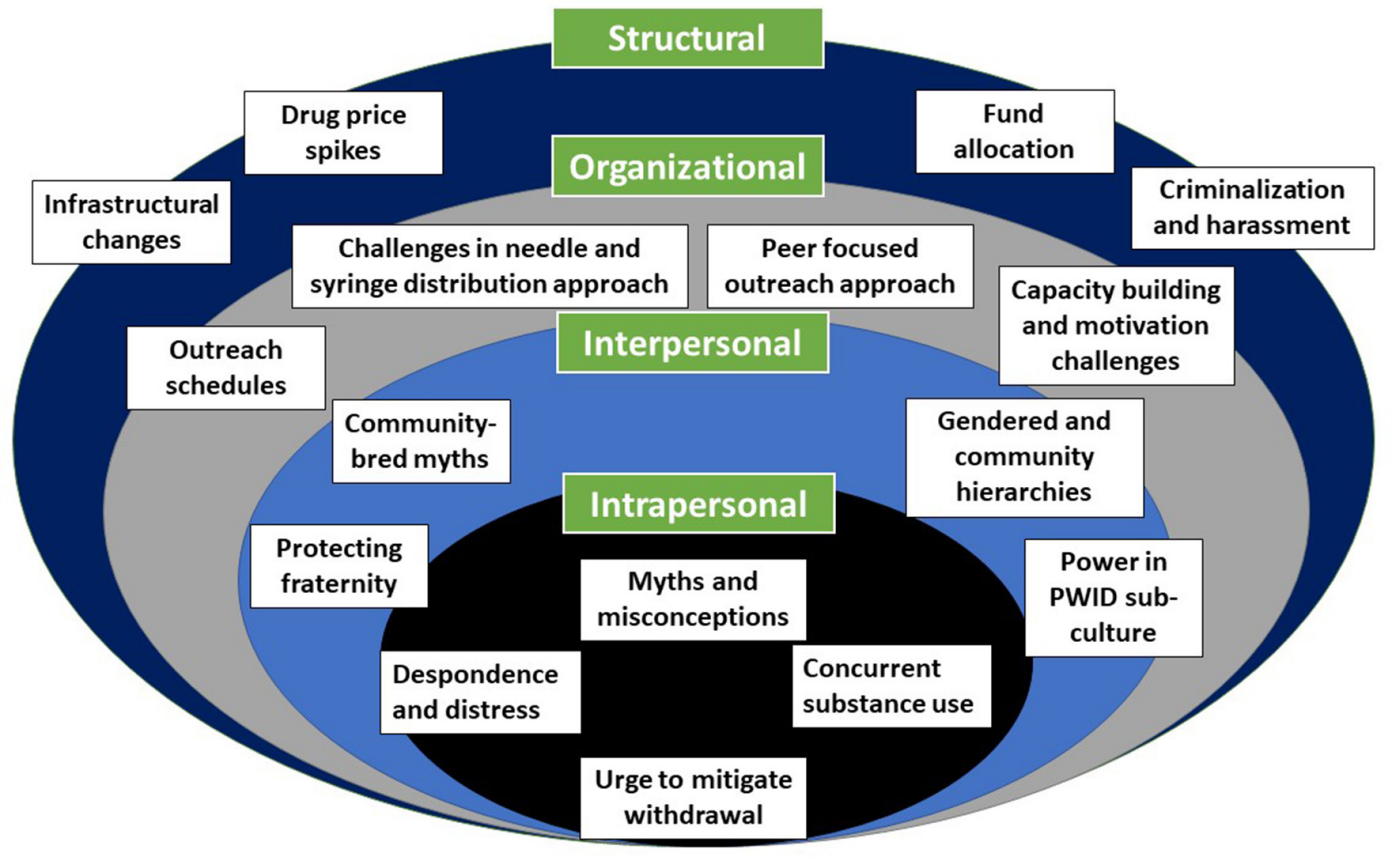
Reasons for needle and syringe sharing as per the socio-ecological framework.

This study explored the contexts of the PWID's socio-structural environments and social locations which affected their HIV risk behaviors, mainly needle and syringe sharing. The findings have been framed in relation to the socio-ecological framework (Figure 1) (49), which include the intrapersonal, interpersonal, organizational, and structural barriers. It is worth noting that these components of the model are not watertight compartmentalized, rather they are fluid and porous, and might intertwine with one another. Our ethnography showed that needle and syringe sharing among PWID not only stems from intrapersonal factors, such as myths and misconceptions regarding needle and syringe sharing, despondence and distress toward their life, concurrent substance use and withdrawal taking precedence over safe injecting behaviors. Interpersonal challenges included the protection of fraternal relationships through sharing, inherited community-bred misinformation about safe injecting practices, influence of gendered and community hierarchies and power dynamics within the PWID sub-culture. Programmatic contexts challenges included inconvenient outreach schedules in relation to PWID's drug-injecting time windows; challenges in the needle and syringe distribution approach; and a predominantly peer-focused outreach approach where peers exhibited work performance and compliance issues, and challenges in capacity building and upholding motivation among OWs. At the structural level, changes in infrastructure and associated inconveniences, criminalization of drug use and harassment of PWID, increased drug prices and financial constraints, and changes in fund allocation policy of the donor engendered risky injection.

### 3.1 Socio-demographic profile of IDI participants

Most of the IDI participants (77.3%) were men, 19.7% were women and 3.0% were transgender women. More than half of the participants (51.5%) belong to 36–60 years of age group. The majority (54.5%) reported not receiving any formal education. Most participants (45.5%) were found to be married, although there were numerous divorced or single participants. The monthly average income was reported as equivalent to $59.5. Participants were engaged in diverse occupations including rag picking, sex work, rickshaw pulling, and petty criminal activities, such as blackmailing and thievery. Among the participants, 39.4% were living on the streets (Table 1).

**Table 1:** Socio-demographic information of the in-depth interview participants.

**Demographic variable**	**Number of participants (%)**
**Gender**	
Male	51 (77.3)
Female	13 (19.7)
Transgender	2 (3.0)
**Age (years)**	
18–25	7 (10.6)
26–35	25 (37.9)
36–60	34 (51.5)
**Level of education (highest)**	
Illiterate	36 (54.5)
Grade 1–5	15 (22.7)
Grade 6–10	12 (18.2)
Beyond Grade 10	3 (4.5)
**Marital status**	
Married	30 (45.5)
Unmarried	16 (24.2)
Divorced/Separated	13 (19.7)
Widower	6 (9.1)
Did not disclose	1 (1.5)
**Average monthly income**	7140 BDT (59.5$)
**Occupation/employment status/primary means of income**	
Unemployed	11 (16.7)
Private sector job	1 (1.5)
Small business	7 (10.6)
Rickshaw or van puller	17 (25.8)
Informal recycling/garbage collecting	8 (12.1)
Sex work	9 (13.6)
Begging/panhandling	4 (6.1)
Petty criminal activities	3 (4.5)
Petty drug seller	3 (4.5)
Cleaner	1 (1.5)
Housewife	1 (1.5)
Other	1 (1.5)
**Living status**	
Floating/Street based	26 (39.4)
Home based	40 (60.6)

### 3.2 Intrapersonal barriers

Findings revelaed that PWID participants shared needles and syringes for various reasons rooted in mental distress, knowledge and awareness about HIV risk behaviors, and personal choices. These contexts were not as heavily influenced by the structural and programmatic factors (described in subsequent sections).

#### 3.2.1 Myths and misconceptions surrounding needle and syringe sharing

Over half of the PWID participants were found to be illiterate or less educated, which influenced their perceptions of safe injecting practices. Notably, we observed that PWID received group educational sessions at DICs and behavioral change communication from outreach workers. Nevertheless, their limited literacy impeded their ability to internalize the importance of the HIV risks associated with needle and syringe sharing. Therefore, despite knowing that HIV can be transmitted through contaminated syringe/needle, they believed that infections only permeated through sharing the same needle, but not the syringe. As one of the PWID opined during an IDI,

“*Only the needle enters the body, therefore, diseases will only spread through the needle. But syringes are plastic so they do not carry disease*.” (Street-based male PWID, IDI)

Our observations also corroborated this phenomenon on several occasions where we noticed PWID changing the needle, but not the syringe.

Moreover, due to these gaps in understanding, they could not fathom the danger associated with HIV, despite being told about the risks and routes of HIV transmission. This is attributed to the perspective that HIV and AIDS did not exude as severe tangible or visible sufferings as other conditions, e.g. injection-related infections, overdose complications, etc. Rather, they claimed that their HIV-positive peers were resuming everyday activities like other PWID. Thus, HIV was not deemed a “big issue”. During an informal discussion within one of our observation sessions, one PWID mentioned that:

“I know some HIV positive PWID but they are doing okay just like everyone else (HIV-negative peers). However, some PWID died because they took the drug into the groin (the needle got displaced and profusely bled). But I never heard of anyone dying because of HIV. This is why many of us are not afraid of HIV, or needle and syringe sharing.” (Street-based male PWID, informal discussion)

#### 3.2.2 Despondence and distress toward life affecting health behaviors

Our findings revealed many PWID were street-based, thus entrapping them within poverty, homeless, food insecurity, and marginalization. These circumstances cemented negative sentiments toward their lives, thus predisposing them to self-reported depression, anxiety and suicidal ideations. Because of their despondence and apathy toward their lives, they disregarded the adverse health consequences of needle and syringe sharing, including the prospect of mortality linked to drug use. According to them, the turmoil associated with HIV would not inflict any additional pain compared to their current state of social exclusion and loneliness. This scenario particularly resonated among PWID who were evicted from their families and lost their scope to generate income in a respectable manner. According to some of the PWID:

People are disgusted with me. They tell me to get lost. My family does not want to see me so they sent me away. People tell me “hey bloody addict, go away”. If I wear dirty clothes and want a glass of water, they kick me out. They say, “Why don't you die”. I am bothered about anything in life now. HIV or AIDS is no issue for me, so might as well share needles and syringes. (Street-based male PWID, IDI)

Therefore, these findings have shown that PWID were entrapped in emotional distress and overall frustration toward their lives to the extent that they no longer deemed safe injecting behaviors a priority.

#### 3.2.3 Concurrent substance use leading to risky injection

The findings captured scenarios where PWID have become more vulnerable to risky drug-taking practices besides needle and syringe sharing. For example, many young and street-based PWID reported concurrently using methamphetamine (locally known as Yaba), alongside injecting buprenorphine. On several occasions, the aggregate effect of opioid withdrawal and *yaba* accelerated the PWID's propensity to relieve their injecting drugs cravings, thus heightening their inclination to share needles and syringes with their peers.

However, our findings also revealed that the concurrent use of methamphetamine elevated their feelings of intoxication, thus predisposing them to impulsive and risky behaviors. In particular, they were observed to partake in illicit money collection mechanisms such as mugging, pickpocketing, touting and drug selling. When they engaged in these activities, they expressed that their state of drug intoxication blurred their perceptions of safe injecting practices, which compelled them to share needles/syringes with their peers. As revealed by the following ethnographic excerpt:

At 9AM, Kabir took methamphetamine with his friend under a blanket near the rail-line. At 10AM in a nearby area, Kabir was running around the railway station snatching passengers' belongings. He then found his friend and shared needles/syringes with him after splitting a drug. After injecting, I asked him why he shared needles/syringes. He explained, “When I am on Yaba, I have no care in the world. In the heat of the moment, I have no time to think of needles and syringes.” (Field diary excerpt-01)

The findings also revealed that some groups of junior PWID who were often dominated by their senior peers needed additional confidence to withstand conflict. Therefore, they perceived the need for additional drugs, believing that these drugs would bolster their audacity and strength, since methamphetamine increased their aggression. This effect increased their propensity to share needle and syringes as it lessened their health-related concerns. As one PWID mentioned during an FGD:

When I take *yaba* I have no care in the world. I feel powerful inside like nothing can hurt me. I can even take down anyone on the street. I no longer think about new or old needles and syringes. I think about new needles/syringes when I am afraid of infection but I am no longer afraid when I take *yaba*. (Male PWID, FGD)

#### 3.2.4 The situation where withdrawal always takes precedence

Withdrawal, a staple of PWID's lives, perpetuted risky injection among PWID suffering from financial hardship and compromised access to needles/syringes. Regardless of their circumstances, their immediate priority was to alleviate their withdrawal. Due to the fluctuating prices in the market and constrained purchasing powers, they had to wait for longer periods to find and inject drugs, thus exacerbating their withdrawal sufferings (elaborated above). In many cases, some PWID felt compelled to share with their peers using readily available needles and syringes because of their reluctance to wait for sterile needles and syringes from outreach workers. As one PWID mentioned during an IDI:

When you are suffering from withdrawal, you feel like dying and want to inject soon. The drug is important, not the type of needle and syringe (new or used). That is why I often borrow used needles and syringes from others. I could have waited for the outreach worker but getting rid of my withdrawal was more important. (Transgender PWID, IDI)

Their withdrawal feelings often clouded their judgment and decision-making abilities, thus predisposing them into the mindset where relieving their withdrawal superseded the risks of needle and syringe sharing.

### 3.3 Interpersonal factors

#### 3.3.1 Protecting fraternal relationships within PWID communities through sharing

The PWID community, mainly the street-based PWID, is closed off from the mainstream society because of the socio-cultural taboos associated with drug use. Since they were ousted from their familial and societal circles, they reported relying on their PWID peers for various forms of support, ranging from financial to moral support. Poor and homeless PWID often relied on peers for drugs, shelter, food, care during illness and adversities, etc. The culture of sharing drugs and needles/syringes exuded the motifs of fraternity and social intimacy, which overruled concerns about HIV infection. As one of the PWID mentioned during an IDI:

The night before Eid-al-fitr (*religious festival of Muslims*), I saw a brother suffering from withdrawal. I had half a drug in my syringe after injection. I told him, “brother, do you need a quarter?” he said, “yes”. Then I injected a quarter from my syringe. He thanked me and I felt really good because I could help someone. We then became very good buddies. (Male Street-based PWID, IDI)

In this community, sharing needles/syringes symbolized emotional intimacy, which blossomed friendships. PWID reported that they tried to preserve these relationships, because a trustworthy friend was perceived as an invaluable commodity (“There are more enemies than friends in the PWID community”) (Male street-based PWID, IDI). Additionally, the PWID not only shared needles/syringes out of a moral obligation to help their peers, but also because they feared losing their friendship in a situation where they were already entrapped in loneliness and social exclusion.

#### 3.3.2 The inheritance of community-bred misinformation about safe injecting practices

Since PWID primarily co-existed within community circles, they acquired community-bred values and norms from peers, which permeated misinformation about safe injecting practices. This dynamic was more pronounced amongst poor and homeless PWID, mainly due to their dependence on their community and low literacy. This included several needle and syringe purification techniques such as licking the needle, flicking or rubbing the needle against a surface, igniting fire, or wiping it with cloth. These practices were believed to eliminate any remnants of contaminant from the previous drug-injecting episode. As one of the PWID reported during an FGD:

My blood-brother (*rokter-bhai*) told me to inject with a used syringe which I clean with a piece of cloth (*gamcha*) beforehand. Sometimes I put water inside the barrel syringe, pump it out to clean it, I pull the piston of the syringe, say my prayers to God and then start injecting. (Male PWID, FGD)

The following field note excerpt during an observation session depicts one of those incidents:

At 8.57AM, I saw two PWID leaning against a roadside divider. They carefully broke the ampoule and loaded the drug into the syringe. One of them injected the drug in his arm andpushed the rest to other PWID. I asked them why they shared the same needle and syringe. One of them replied, “Brother, I heard he is HIV positive and I am positive too. We both have the disease. If we share, nothing will happen.” (Field diary excerpt-02)

#### 3.3.3 The influence of gendered and community hierarchies on risky injection

PWID communities often relied on each other for various forms of support, including injection assistance. Our observations revealed that many inexperienced PWID were scared of injecting drugs independently. In these instances, more experienced, male and senior or financially solvent members of the PWID community would assume ownership over the drug-injecting skills and equipment. Hence, newer PWID could not negotiate safe injection, thus fueling their needle and syringe sharing behaviors. The following observation note from a PWID shadowing session illustrated this dynamic:

I was strolling along a popular spot in a morning and saw a PWID who just bought drugs and searched for his senior peer to help him push the drug into his leg. The peer told him that he is willing to help inject in exchange of money for pushing drugs. So his peer injected on his shoulder using an already used needle and syringe. (Field diary excerpt-03)

This dynamic was widely observed among female PWID where their male partners assisted in their injection, often at the expense of their agency over safe injection. Due to the unequal gendered power differentials, female PWID lacked the scope to negotiate using sterile needles/syringes out of anticipatory fear of gender-based violence. For many female PWID, IDIs revealed that their male intimate partners were considered their safety net, providing shelter and protection from unwarranted sexual harassment. Therefore, going against their male partners' demands not only perpetuated violent situations but also incurred threats of being evicted from their homes and/or partnerships.

#### 3.3.4 Power dynamics within the PWID sub-culture

Our findings revealed social hierarchies within the PWID community. Apart from economic solvency, the PWID‘s social capital (i.e., connections within and outside the community), physical strength, domineering attitude, etc. defined their hierarchal positions. For example, some local PWID who had connections with local socio-political power structures (i.e. politically influential people and some members of law enforcement agencies) and resided at the spots for several years assumed powerful positions. Conversely, many PWID who lacked those connections were subjected to dominance. This power imbalance affected various facets of their drug-injecting behaviors including the quantity of drug intake, order of injecting and needle and syringe sharing. Since some influential PWID were inclined to exercise their authority through intimidation, their powerless peers felt compelled to succumb to their demands. One of the male HIV-positive PWID rationalized his practices in the following way during an FGD:

One day, a powerful PWID passed by me and could not find drugs. He saw me loading drugs into the syringe and I considered injecting half and saving the other half for later as we were in a crisis. But he demanded half of the drug. I was so scared, I gave him my drug and syringe, otherwise he would snatch my money. (Male HIV positive PWID, FGD)

If the PWID did not comply with their demands, they were often subjected to various adversities such as physical harassment, extortion, eviction from the spots, blackmailing, etc. In this context, subservient PWID were found to follow these requests in exchange for survival.

### 3.4 Organizational factors

Our study findings presented scenarios where the harm reduction programs (i.e., the needle and syringe program (NSP) for PWID) faced challenges which posed difficulties for constraining needle and syringe sharing practices. This elevated the risk of HIV transmission. This section delineates outreach design and implementation challenges.

#### 3.4.1 Outreach schedules in relation to PWID's drug-injecting time windows

**Figure 2:**
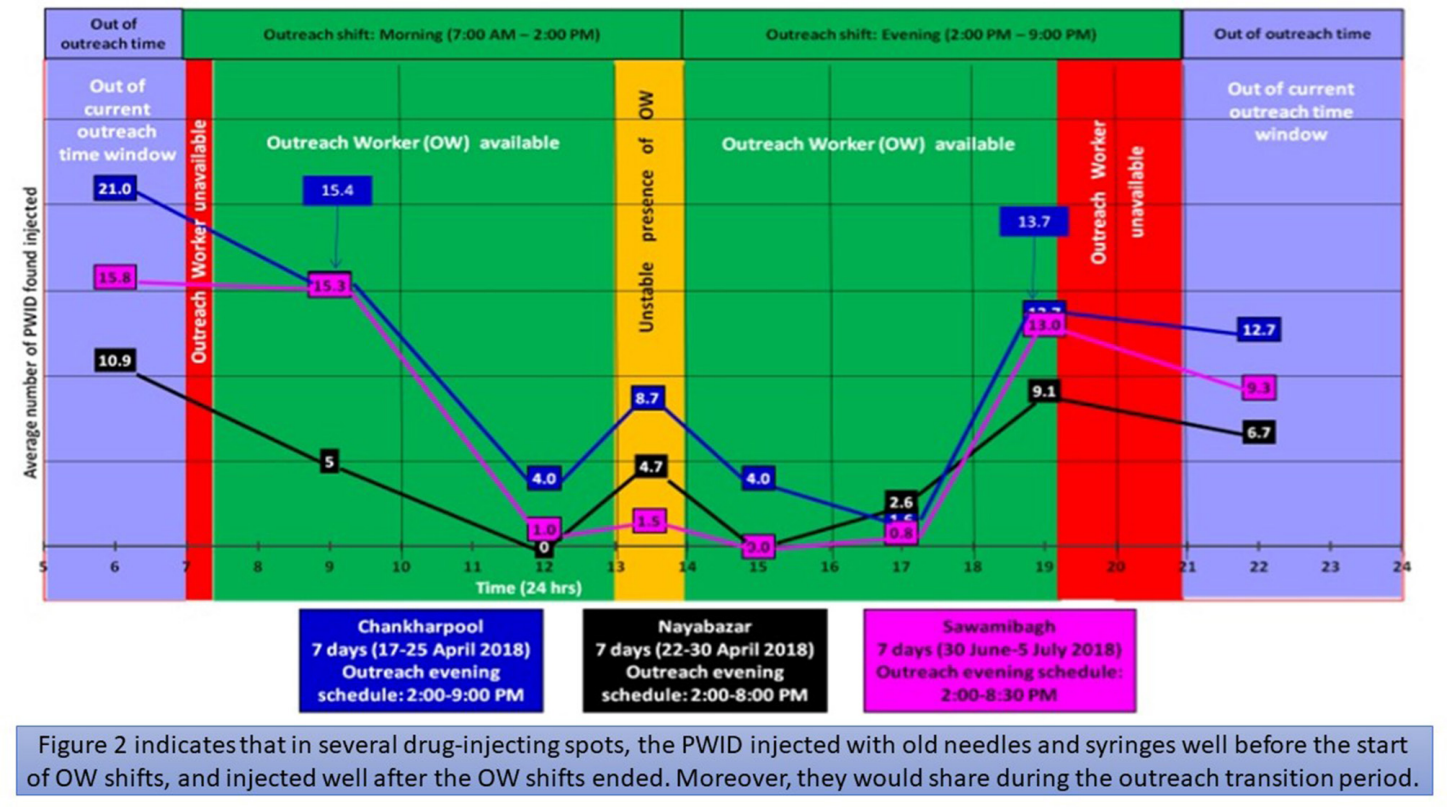
Visibility of PWID in different timeframes at three drug-using spots and avaiaiblity of OWs.

We conducted 30 early morning observation sessions at multiple drug-injecting spots. These sessions revealed that PWID would often inject drugs as early as 5:30 AM (Figure 2), but there were no early morning shifts available during the study period. Numerous PWID, including HIV positive PWID, injected drugs in the spots from 5 to 7AM. As needle and syringe outreach services were not available before 7AM, PWID struggled with managing needles and syringes, as pharmacies were closed. These situations left PWID to borrow needles/syringes from their peers. According to a field diary excerpt:

At 6:30AM, Mamun and his drug-injecting partner, Mohon, bought an ampoule together. However, Mohon did not have an extra syringe. Outreach shifts did not start until 7 AM. Yet, the withdrawal was so unbearable that they could not wait any longer so they shared. (Field diary excerpt-04)

Furthermore, during the data collection period, outreach services were not available after 8-9PM even though 31 observation sessions indicated that many PWID congregated at the spots after that time window (Figure 2). Therefore, PWID who could not collect needles and syringes beforehand relied on their peers for needles and syringes, which ultimately compelled needle and syringe sharing. During an observation session, one of the PWID mentioned that:

I am a rickshaw puller and I usually work until 10PM if not later. Before I head back home, I usually stop by the spot to take drugs. Most of the time, I have to collect needles and syringes from other druggies (*neshakhor*) but some of these needles are old. But I have no other choice but to use them. (Home-based male PWID, Informal discussion)

We conducted observations from the early morning to the afternoon for 30 days and and 31 days of evening observations at three major spots (Figure 2). Our observations included 32 shadowing sessions with the outreach workers (OWs). Findings illustrated that OWs arrived ~59.2 min late, on average. This led to spots being vacant at times when PWID needed sterile needles and syringes. Figure 2 also depicted that OWs left their shifts at least an hour and 22 min earlier than their assigned times, on average. After outreach shifts ended in the evening, 7 to 13 PWID were found to be present at the spots. In addition, 2 to 11 PWID were observed to inject drugs during the transition period between the morning and the evening shifts (1–2 PM). However, they were unable to obtain needles and syringes during those times because the PWID reported that some OWs left their morning shift early and/or arrived late to their evening shifts (Figure 2). As one of the PWID mentioned during an IDI:

I need to get out of my house in the morning and go to the spot to collect needles and syringes. However, this is often not possible because the OWs come late. I can't just sit and wait around. I might as well share with other PWID instead. (Home-based male PWID, IDI)

Moreover, observation findings identified that outreach services were relatively lenient during weekends and public holidays, despite provisions for administering outreach services during those times. According to observations from 12 Friday sessions and six public holiday sessions at five major spots, all OWs arrived late to their respective duty stations and took mid-day breaks more frequently during their assigned shifts. The following field diary excerpt, which occurred during a weekend observation session, depicted such instances:

I shadowed the OW over the weekend. He wanted to take a tea break. At 10:15 AM, a drug peddler arrived and sold an ampoule to four PWID. They started looking for the OW but after waiting for a long time, they were vilifying him. They found a needle on the footpath, thinking it was new (but it looked old) so they shared that. (Field Diary excerpt-05)

#### 3.4.2 Challenges in the needle and syringe distribution approach

One of the key challenges that the NSP faced was the implementing the needle and syringe distribution approach at the outreach spots, as there were no written guidelines for needle and syringe distribution during the study period. This bred ambiguity and contradictory interpretations of the DIC management officials' verbal instructions. 50 shadowing sessions indicated that PWID were not always receiving the desired number of needle and syringes (exemplified in the below excerpt). Consequently, PWID felt compelled to use and share borrowed syringes from their peers.

I heard an OW arguing loudly with a PWID because he was asking for two syringes but the OW did not want to give more than one. The PWID was trying to explain that he and his friend are going to share an ampoule therefore they needed two separate needles and syringes but the OW did not listen. After the PWID stormed off, he loaded the drug in the syringe and shared the same syringe with his friend. (Field diary excerpt-06).

Moreover, the DICs often struggled to distribute the preferred needle and syringe sizes. Two sizes of needles were available, i.e. 23 gauge (23 g) and 27 gauge (27 g), which are big and small needles, respectively. Syringes were available in two sizes, i.e. 5cc and 3cc syringes. The latter size seemed favorable because of its perceived ability to minimize drug wastage since they were narrower than 5cc syringes. However, since DICs distributed equal amounts of 3cc and 5cc syringes and 23g and 27g of needles, there was a demand-supply gap of 3cc syringes. Likewise, PWID preferred 23g needles because they perceived that these needles would accelerate the transmission of the drug into the bloodstream. However, since 23g needles were higher in demand, this engendered the same demand-supply gap. The following field diary excerpt illustrates this phenomenon:

At 12:30 PM, I saw the OW and field monitor arguing. The OW exclaimed, “I'm out of 23g needles, where am I going to get more needles from?” The whole stock was exhausted, except for some 27g needles that PWID did not want. Later, some PWID were looking for 23g needles from other PWID so they ended up sharing. (Field diary excerpt-07)

#### 3.4.3 A predominantly peer-focused outreach approach

During the study period, 80% of the OWs were from the PWID community. Although the peer OWs were able to quickly identify and access covert drug-injecting locations and PWID, they had some limitations attributed to their drug-using behaviors. Specifically, peer OWs commonly demonstrated poor punctuality e.g., late arrival and early departure from the field (Figure 2) and their long-term drug use affected their stamina and mental fortitude. PWID also expressed their inability to seriously internalize the OWs' advice because it exuded hypocrisy. According to 32 shadowing sessions with OWs, several peer OWs needed to take drugs before starting outreach shifts to gain energy for their work. Observation and interview findings revealed that many peer OWs were less inclined to comply with office rules and more likely to take unauthorized leave than non-peer counterparts. They were also shown to exhibit aggressive attitudes toward management staff because of disputes about punctuality issues. As one of the DIC coordinators in a KII described:

Peer OWs have to take drugs before their shift. They come to the field at 9AM but were supposed to come at 7AM. If they can't find drugs, they come to the office even later. Some of them are also OST clients and spend around 1 to 2 hours just for OST. Because of this, the field remains vacant. They are all Bill Gates, Obama and the UN Secretary-General. It is hard to say anything to these people. (DIC coordinator, KII).

Moreover, our findings encapsulated scenarios where peer OWs limited themselves to one spot or specific area within a spot despite being assigned to work in multiple areas, leaving many spots unattended. Under these circumstances, some withdrawal-ridden PWID were left with no viable option to attain sterile needles and syringes. As one of the PWID explained during an FGD:

They sit in one place all day long without moving around to find us. They are supposed to walk around and give us syringes. Sometimes you will see them sitting far from the spot or at a tea stall. When we are suffering from drug withdrawal (berar shorire), we do not feel like walking. We will inject with whatever is around us even if it's a borrowed needle. (HIV positive PWID, FGD)

#### 3.4.4 Challenges in capacity building and upholding motivation among OWs

KIs claimed that they recruited willing and eligible PWID in the community as peer OWs. However, field-level findings stipulated that, due to time constraints, OWs were primarily recruited on an *ad hoc* basis to upkeep the substantial turnover rate. In most cases, the recruited peer OW is not always part of the same locality as their enlisted PWID. As a result, the main advantage of engaging peer OWs was not being leveraged, i.e. utilizing pre-existing peer rapports to facilitate access to PWID communities. During the data collection period, we identified eight newly recruited peer OWs at three DICs who reported having no formal orientation before their deployment. Consequently, it took them considerable time to learn appropriate outreach service delivery approaches, and understand spot dynamics.

A substantial portion of the OWs complained about their meager salaries during the observation sessions, which they deemed insufficient for their sustenance, thus exacerbating their frustration and lessening their motivation. On some occasions, informal discussions revealed that they lacked the motivation to attend their duty stations on time. These circumstances were cumulatively observed and reported to underpin a risk environment for PWID to share needles and syringes because they were not receiving the necessary services.

### 3.5 Structural factors

Observation and interview findings showed that PWID were considerably affected by environmental, socio-political, and legal influences, which propelled risky injecting behaviors.

#### 3.5.1 Changes in infrastructure and its associated inconveniences

Due to rapid urbanization and infrastructural modernization, Dhaka saw an increase in population size and infrastructural developments. Amidst these circumstances, numerous drug-injecting spots e.g. parks, abandoned street corners, dilapidated buildings, etc. were either closed down by authorities or defunct after infrastructural development activities increased. Since many PWID were poor and homeless, there was no scope for them to inject safely in an enclosed shelter. Moreover, OWs could not reach them for rendering educational support. As mentioned by a DIC staff member during a KII:

Some changes happened like new high-rise buildings, roads, flyovers, alleyways. This forced PWID to inject at easily hidable places. The spots used to be in open spaces and outreach staff could easily see PWID and reach them for needles and syringes. But this is not possible now, so PWID are sharing their needles and syringes. (DIC manager, KII)

#### 3.5.2 Criminalization of drug use and harassment of PWID

As per the existing narcotic control law, possession, carrying and use of drugs are considered criminal offenses in Bangladesh (GoB 2018), thus subjecting the PWID to law enforcement interference, harassment and, sometimes arbitrary arrests. Findings from repeated observations (including shadowing) at multiple spots indicated frequent spot raids by the law enforcement forces during, spanning up to 5 h. Many PWID, especially street-based PWID, escaped, migrated to nearby cities, and scattered around the city out of fear of frisking, interrogation, and confiscation of drugs and needles/syringes. This impeded the PWID's ability to visit the spot to attain sterile needles/syringes. Since outreach workers were responsible for distributing needles/syringes, they were often susceptible to law enforcement harassment and interrogation, under the allegation that they were enabling drug use. These incidents invoked fear and apprehension, thus compelling PWID to share needles/syringes despite knowing the risks attached to sharing. As one of the PWID mentioned during an IDI:

You need to strategically avoid getting caught by the police. If caught, the police will not only harass you but will also snatch your drugs, syringes, money, and other valuables and throw you in jail. Since we are so careful in trying to avoid them, we have no luxury to consider sterile needles/syringes. (Street-based male PWID, IDI)

#### 3.5.3 Increased drug prices and financial constraints

Managing money for drugs was highlighted as a key struggle underpinning the PWID's lives. As relayed by Table 1, most of them held odd jobs, thus lacking steady sources of income. Their financial constraints compromised their purchasing powers; thus they could not upkeep the volatile drug market. We observed that drug prices fluctuated frequently and sometimes increased exponentially, sometimes on the same day. Key-informants reported that drug dealers maintain a nexus between local politico-legal power structures. If any dissonance arises in this understanding, law enforcement agencies are more likely to stringently impose drug seizures, thus heightening the demand-supply gap and leading to price spikes. This situation also highlighted the dynamic where some PWID were at a comparative disadvantage of procuring affordable drugs because of their poor purchasing powers and lack of socio-political connections. Consequently, PWID who were formerly willing to buy a whole ampoule would buy fractional increments instead. Since many of these drugs were shared among multiple PWID, this inevitably caused needle and syringe sharing. A CBO leader mentioned during a KII:

There is a shortage of drugs which explains the steep prices. Therefore, the PWID cannot afford it themselves and have to share drugs with others, which often involves needle and syringe sharing. Since PWID think that changing syring will waste their drugs. (Community-based organization leader, KII)

Drug prices peaked during the anti-drug drive. In that context, many PWID with poor purchasing powers often relied on buying quarter or less than a quarter of an ampoule from fellow PWID, thus increasing their chances of needle and syringe sharing. Many PWID, who injected a quarter of half of the ampoule, expressed concern about wasting drugs in the process of dividing the drug into multiple syringes, thus increasing their inclination to share. Following a conservative approach was deemed to be the most viable option because of their modified social location in the economic domain. As one of the PWID mentioned during an IDI:

Yesterday the drug price was 800 taka ($9.50). One-quarter of the drug (0.5 ml) cost 200 takas ($2.40). If we divide it into four syringes, we will lose 1-2 drops of drug because the needle consumes some drug everytime it is pulled from the ampoule. One drop costs around 40 taka ($0.50). We cannot afford to waste 40 taka ($0.50). The last person who will pull the drug from the ampoule will lose one drop and he will never accept that, he may even fight. Might as well share. (Street-based PWID, IDI)

#### 3.5.4 Changes of the fund allocation policy of the donor

We found that HIV prevention interventions in Bangladesh, which were supported by the Global Fund since 2008, cycled through different funding installments which ultimately shaped various facets of the harm reduction program. The most drastic fund cut was documented in 2015–2016, thus illustrating a 54.8% fund reduction during the transition between two funding cycles. The program-implementing stakeholders claimed that, due to fund reduction, they had to economize by reducing PWID coverage, decreasing the number of DICs (17 to 11) in Dhaka, increasing the ratio of PWID to outreach worker (40:1 to 72:1), reducing the number of needles and syringes distributed to PWID per year (337/year versus 555/year), and reducing field monitoring staff. They claimed that the syndemic effects of these changes increased needle and syringe sharing.

On the other hand, some key-informants opined that needle and syringe sharing cannot be solely attributed to fund cutting. They posited that PWID interventions changed on the basis of fund cuts, rather than based on evidence or consultative discussion with expert stakeholders. Moreover, since the HIV prevalence was low for a long time, program stakeholders were complacent in believing that the interventions were fulfilling their agenda of reducing HIV infection. According to a key-informant, “adequate funding is important, but considering the scenario of changing funds, programmatic innovations are necessary so that the design can accommodate major programmatic components even when funding is low” (Program expert, KII).

## 4 Discussion

This study adopted a PEER approach framed by the socio-ecological model to understand risk contexts of needle and syringe sharing among PWID to the socio-ecological model explored the role of PWID's multilayered risks. In Bangladesh, limited research was conducted on HIV risk behaviors among PWID, including a qualitative study in northwestern Bangladesh (26, 27). This is one of the first studies in the region to perform a PEER analysis to explore the lived experiences, practices, community dynamics, and risk behaviors of PWID. Amidst the existing evidence gap about the programmatic (organizational) challenges, this ethnographic analysis bridged this gap by providing insights for strengthening NSPs in Bangladesh and other similar settings.

The individual layer of the socio-ecological model encapsulated the individual struggles, emotions, beliefs and perspectives of needle and syringe sharing, which remained underexplored in other research. Our findings highlighted PWID's apathy and despondence toward their lives, thus overruling concerns about the implications of needle and syringe sharing. Though similar sentiments resonated in other studies (27, 50, 51), the role of poverty and social exclusion not yet explored to this degree. Our findings also illuminated the link between concurrent use of methamphetamine and risky injecting behaviors. A few studies alluded to poly-drug use influencing needle and syringe sharing (52–55). However, our study also identified new dimensions of concurrent drug use including community-based violence and power dynamics.

Risky injecting practices were attributed to misconceptions about safe injecting, linked to both intrapersonal and interpersonal circumstances. Since PWID in our study were mainly confined within their street-based communities, most learnings and perspectives were inherited from peers, including injecting and equipment purification practices. Although a few studies indicatedlimited purification methods for used needles/syringes (26, 56), our study evidenced new unchallenged misconceptions of “cleaning” the needle and syringe such as igniting a fire or wiping with cloth. Since these practices originated from community-based norms, it is crucial to engage community-based organization leaders to debunk these misconceptions, and enhance behavior change communication within harm reduction programs.

Our findings at the interpersonal layer also illustrated that PWID sought community support systems due to societal marginalization. This often perpetuated risky injecting behaviors, such as seeking injection assistance from peers. This phenomenon resonated among female PWID in a few other studies (57, 58). Our study highlighted another dimension of community interdependence, which involved PWID succumbing to peers to sustain interpersonal relationships. Although the motif of fraternity was deliberated in other literature (27, 59), theoretical exploration was lacking. Moreover, the notion of power hierarchal dynamics was reflected in our study where seniors dominated juniors, thus fueling needle and syringe sharing. Similarly, Bourgois and Schonberg depicted racialized power structures between black and white PWID, socio-political barriers and its relationship with needle and syringe sharing behaviors was not explored (30). Likewise, a recent qualitative study in Iran revealed that new, inexperienced PWID were likely to lean on senior peers as opposed to DICs (59), necessitating demand generation for harm reduction services.

Our study revealed various programmatic design and implementation challenges, which made it difficult to avert sharing behaviors of PWID. For example, the observations and shadowing sessions alluded to the inopportune mismatch of outreach schedules based on PWID injecting times, which was also corroborated by other studies (60–62). However, our study leveraged ethnographic observations and anecdotes to illuminate the effects of programmatic limitations. Since it is not always programmatically feasible to incorporate early morning one-to-one services, other innovations need to be considered (described in implications of findings section).

Our observations also pinpointed the needle and syringe access challenges that emerged from an overly peer-focused outreach approach. Several other studies criticized the reliability of the OWs (mostly peer OWs) for their punctuality (62–64). Similarly, a study based in China evinced that peer OWs could exhibited poor work performance and punctuality (64). Our repeated observations also revealed relaxed services during weekends and public holidays, showing NSP's inability to continuously meet PWID's needs for needles and syringes across different timeframes. However, such similar evidence remains limited. In this context, our study implicated the importance of closely monitoring drug activity during holidays and tailoring outreach services accordingly. Observation sessions and PWID anecdotes found that the OWs stuck to a single area without branching out to other areas, thus exacerbating service access barriers. Although limited movements were also highlighted in other studies, those studies merely attributed this limitation to a lack of funds to cover transportation costs (65) but did not further investigate how these movement limitatons propagated risky injection.

Our findings also highlighted challenges in the needle and syringe distribution strategy. This could potentially compromise needle and syringe uptake for PWID. This was yet to be found in other studies because OWs were not directly inquired about their understanding of the needle and syringe distribution approach. Furthermore, this study evinced that 3cc syringes and 23g needles are more popular than 5cc syringe and 27g needle, engendering a demand-supply gap. Similarly, a study in Tajikistan revealed that the most popular syringe sizes were 2cc and 5cc and the preferred needle sizes were 21–25 gauge (66).

At the structural level, we also explored the implications of legal and financial policies on needle and syringe sharing. Findings suggested that intensified law enforcement activity influenced needle and syringe sharing behaviors. Previous literature revealed risky behaviors and adverse events such as increased needle and syringe sharing, decreased uptake of outreach services, increased HIV/Hepatitis-C virus (59). Our study also portrayed new insights about needle and syringe sharing emerging from law enforcement activity such as haphazardly injecting to avoid harassment, and dispersing to remote, underserved areas, which were not covered in other research. Thus, law enforcement agencies need to be sensitized by facilitating advocacy sessions and redesigning their training curricula to accommodate HIV-related issues. Moreover, relevant ministries need to be actively involved in revising some legal clauses. Implementers of the programs we worked with also found that reductions in funds engendered increases in needle and syringe sharing. A study in Iran briefly cited the link between poor budget allocation for DICs and needle and syringe sharing (67) but did not articulate the exact component-wise pathways which precipitated needle and syringe sharing.

In our study, we found that rapid urbanization and infrastructural development stripped poor and homeless PWID of their scope to uptake sterile needles and syringes. The motif of housing instability was also reflected in other recent research (68, 69). Our findings also indicated the PWID's low purchasing powers amidst situational price spikes, thus compelling them to share needles and syringes. Although the relationship between money and sharing was corroborated elsewhere (59, 70, 71), our ethnography revealed the instantaneous (i.e., sometimes hourly) fluctuations in drug prices, thus portraying how the volatile drug market constrained the PWID's purchasing powers and needle and syringe sharing.

### 4.1 Implications of findings and recommendations

Based on this analysis, individual risk reduction interventions may not be able to adequately reduce risk behavior and alleviate HIV epidemics. Rather, it is integral for the existing harm reduction interventions to account for the multifaceted contexts constructing the PWID's needle and syringe sharing practices. Therefore, harm reduction interventions need to strive toward a paradigm shift which focuses on the PWID's socio-structurally rooted circumstances. In Bangladesh and other settings which are experiencing similar epidemics, it is crucial for harm reduction interventions to analyze their respective programmatic design and implementation gaps, tailor them accordingly and apply holistic intervention with innovations. To improve needle and syringe access, harm reduction programs could implement practical solutions such as mobile distribution points, vending machines, and improved capacity strengthening for OWs.

While some barriers could be improved by adjusting the NSP, others warrant broader policy changes. Barriers which could be resolved through NSP design and implementation modifications include: dispelling myths and misconceptions; counseling for mitigating mental distress and concurrent substance use; withdrawal management; group and couple counseling sessions to avoid the harms of hierarchal community and intimate partner dynamics; and adjustments in the outreach and needle and syringe distribution strategy. Whereas, the structural barriers (e.g. criminalization of drug use, drug economies, and fund allocation policies) need to be alleviated through legal and policy reform. Moreover, initiatives could be adopted to advocate for the decriminalization of drug use, reminiscent of a similar model in Portugal (72). In this model, a stepped care approach is used where low risk cases are not intervened, moderate risk cases are referred to brief interventions (e.g. counseling) and high risk cases receive non-mandatory referral to specialized treatment services (72).

### 4.2 Study strengths, limitations, challenges and future directions for research

One of the major strengths of this study is the application of the PEER ethnographic approach. Our study possessed the overall strength of rigorously blending diverse qualitative data collection techniques (shadowing, walk-and-talk, informal interviews, IDIs, FGDs and KIIs). We had prolonged engagement with the field, irrespective the time, day, and location. Moreover, we leveraged the lived experiences of the PWID community by deploying peer researchers from the beginning of data collection to data analysis and report writing. However, this study carried a few limitations. For instance, since the research team consisted of both peer and non-peer researchers, observations of non-peer researchers may have influenced the injecting behaviors of some PWID due to social desirability biases. However, the researchers overcame this barrier through prolonged engagement and building rapports with the PWID community. Moreover, since ethnographic insights are tied to particular cultural, spatial and temporal settings, this may affect the transferability of all findings to other contexts. One major phenomenon that could not be reached with this ethnographic design was the analysis of quantifiable causal relationships. A future quantitative or mixed-methods study could be conducted to examine the burden and correlates of such phenomena. While it was possible to identify risk contexts of needle and syringe sharing, proving the factors that increase the likelihood of sharing would demand statistical analysis. Therefore, future research could adopt quantitative approaches.

Moreover, data collection challenges emerged throughout the study period. From early May 2018, a nationwide anti-drug drive known as “war on drugs” was declared, which disrupted regular observations for a couple of weeks, due to police raids and an overall climate of fear toward law enforcement interference. Because of this, PWID and OWs were less likely to come to the field. Additionally, due to a political unrest and general election in December 2018, similar patterns of law enforcement interference were observed, thus interrupted data collection for a few weeks.

## 5 Conclusion

Using a peer-driven ethnographic methodologies and theoretical proposition, the findings of this study explored various contexts for needle and syringe sharing among PWID, which are rooted in interlocking systems of diverse socio ecological contexts. Relevant stakeholders and policy planners may need to tackle these socio-structural issues by modifying the programmatic design and implementation approaches, not only in Bangladesh, but in other similar social contexts. Individual risk reduction approaches alone cannot sufficiently contain needle and syringe sharing, nor can the mere application of traditional structural interventions which are limited to only a few advocacy/sensitization efforts. This article demonstrated that the diversified issues, ranging from legal, socio-political and economic issues, are often not addressed by the conventional harm reduction model, thus quite often fail to contain sharing. To reduce HIV among PWID, programs must go beyond information and supplies. They must also address the social and structural barriers that shape everyday choices.

## Data Availability

The datasets presented in this article are not readily available because restrictions are made to the data as per the data policy of icddr,b. The data is also strictly confidential due to the sensitive nature of the populations and the criminalization of drug use as per local laws. However, data can be made available upon reasonable request. Requests to access the datasets should be directed to shiblee_s@icddrb.org.
